# Evaluating the Health Impact of Large-Scale Public Policy Changes: Classical and Novel Approaches

**DOI:** 10.1146/annurev-publhealth-031816-044208

**Published:** 2017-03-20

**Authors:** Sanjay Basu, Ankita Meghani, Arjumand Siddiqi

**Affiliations:** 1 Centers for Health Policy, Primary Care and Outcomes Research; Center on Poverty and Inequality; and Institute for Economic Policy Research, Stanford University, Stanford, California 94305; 2 Department of Medicine, Stanford University, Stanford, California 94305; 3 Center for Primary Care, Harvard Medical School, Boston, Massachusetts 02115; 4 Department of Epidemiology and Department of Social and Behavioral Health Sciences, Dalla Lana School of Public Health, University of Toronto, Toronto, Ontario M5T 3M7, Canada; 5 Department of Health Behavior, Gillings School of Global Public Health, University of North Carolina, Chapel Hill, North Carolina 27599

**Keywords:** difference-in-differences, propensity score, synthetic controls, regression discontinuity, instrumental variables, near-far matching

## Abstract

Large-scale public policy changes are often recommended to improve public health. Despite varying widely—from tobacco taxes to poverty-relief programs—such policies present a common dilemma to public health researchers: how to evaluate their health effects when randomized controlled trials are not possible. Here, we review the state of knowledge and experience of public health researchers who rigorously evaluate the health consequences of large-scale public policy changes. We organize our discussion by detailing approaches to address three common challenges of conducting policy evaluations: distinguishing a policy effect from time trends in health outcomes or preexisting differences between policy-affected and -unaffected communities (using difference-in-differences approaches); constructing a comparison population when a policy affects a population for whom a well-matched comparator is not immediately available (using propensity score or synthetic control approaches); and addressing unobserved confounders by utilizing quasi-random variations in policy exposure (using regression discontinuity, instrumental variables, or near-far matching approaches).

## INTRODUCTION

“Politics is nothing else but medicine on a large scale.”

— Rudolph Virchow

Public health researchers have long asserted that social, economic, and environmental determinants of health may be addressed through large-scale public policy interventions (79). In particular, fiscal policies (e.g., tobacco taxes), regulations (e.g., sanitation standards), and social safety net programs (e.g., poverty reduction programs) have been thought to produce important benefits for population health (24, 27, 34, 65, 72).

For public health researchers, however, a key concern has been the attribution of causality: Does the policy produce the health effects observed, or are the health effects caused by some confounding factor? For example, were lower rates of myocardial infarction (2) due to New York City’s ban on *trans* fats, because of better health care coverage, or because of another factor? Similarly, did California’s Proposition 99 (29), which increased tobacco taxes and banned smoking in workplaces, significantly lower smoking-related disease in the state, or was the decline merely a continuation of preexisting cultural trends?

Many public health interventions, particularly those at the individual or the household level (e.g., dietary interventions), rely on randomized controlled trials (RCTs) to balance the bias of unknown variables. However, practical, budgetary, and ethical barriers prevent large-scale policy changes from being evaluated through RCTs. For example, it would be challenging (if not comical) to randomize some New Yorkers to eat at restaurants where *trans* fats were banned and randomize other New Yorkers to eat at restaurants where *trans* fats were not banned.

Evaluating large-scale policies through classical cohort or case-control study designs poses related challenges. Exposure to the policy may not be random because a policy may specifically target a group for its higher disease risk (producing confounding by indication) (70). Hence, the resulting differences between the exposed and unexposed populations may not be from the policy alone but from population differences or selection bias. One of the most challenging differences to address between populations are unobserved confounders—unmeasured factors that influence both the policy of interest and health outcomes among the exposed group, distorting the true relationship between exposure to the policy and new health outcomes. Consideration of unmeasured confounders becomes especially important when policies are more likely to be enacted in some communities than in others. For example, has California’s tobacco smoking rate been lower than Nevada’s smoking rate because of California’s strict tobacco control policies, or because Californians are more health conscious, and therefore both more likely to vote for an anti-tobacco ballot proposition and less likely to smoke even without the policy in place?

To address these challenges, public health researchers have leveraged several observational data analysis approaches that are derived mostly from the fields of economics, sociology, and political science (43, 80). In this article, we review classical and novel analytic approaches that have been adopted in the public health literature and have been applied to the task of evaluating the health consequences of large-scale public policy changes. Our discussion is organized into three themes that emerged in our review as key challenges faced by public health researchers who analyze policy changes: (*a*) distinguishing a policy effect from time trends in health outcomes or preexisting differences between policy-affected and -unaffected populations; (*b*) constructing a comparison population when a policy affects a population for whom a well-matched comparator is not available; and (*c*) addressing unobserved confounders.

## REVIEW STRATEGY

We performed a PubMed database search in December 2015 to search across all available years for the Medical Subject Heading (MeSH) “Policy” and for original research articles with “Publication Type” listed as evaluation studies, reviews, cost-effectiveness studies, or meta-analyses. This search yielded 1,021 articles, and we applied the following criteria to them for inclusion in our review: (*a*) The article evaluates one or more policy interventions, which we defined as regulations, laws, fiscal policies, or mandates that influence a large population through a government authority at any level (e.g., county, state, nation, international treaty); (*b*) it incorporates a quantitative assessment of policy effects on one or more health risk factors or outcomes; and (*c*) it is not exclusively a commentary, letter, or theoretical simulation. We focused our review on public health studies in which a policy change occurred in a population and the researchers sought to examine the effect of the change on a change in health outcomes. Hence, we excluded studies that examined only fixed differences in policies and health status among groups, such as cross-national ecological comparisons of wealth inequality and mortality.

Thirty-nine articles met our inclusion and exclusion criteria. We first assessed the articles by populating a prespecified spreadsheet with information on study year and duration, policy evaluated, method(s) of evaluation, study challenges/limitations, and key findings. We subsequently classified articles into one (or more) of three categories: (*a*) distinguishing policy effect from time trends or preexisting differences, (*b*) constructing a comparison population, and (*c*) addressing unobserved confounders. For each category, we created subcategories on the basis of the methods we found commonly applied by the researchers: difference-in-differences approaches (under category *a*); propensity score and synthetic control approaches (under category *b*); and regression discontinuity, instrumental variables, and near-far matching approaches (under category *c*). Primary methodological references cited in each article were also reviewed to supplement our analysis, as further noted below.

## DISTINGUISHING A POLICY EFFECT FROM TIME TRENDS OR DIFFERENCES AMONG COMMUNITIES

**Figure 1:**
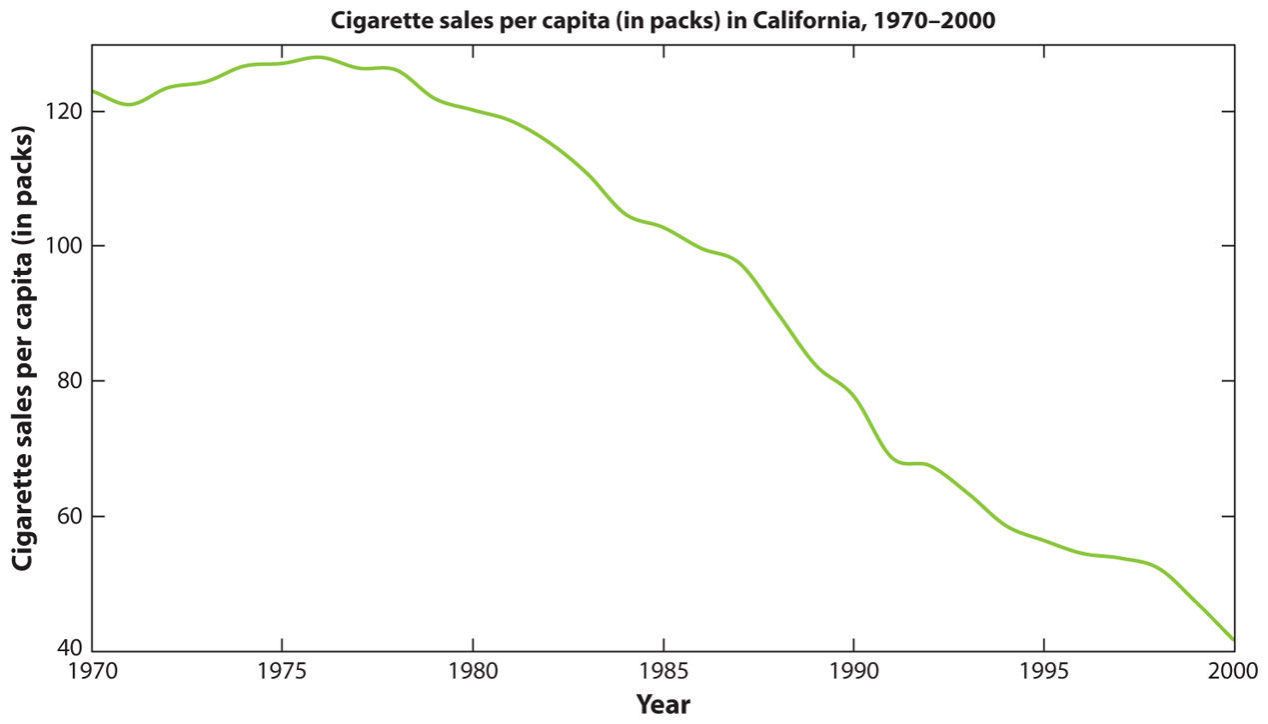
California’s cigarette sales per capita. Proposition 99, which brought forth new tobacco taxes and clean air laws, was implemented in 1988. A problem with simple pre-post analysis is that cigarette sales were already declining prior to implementation of the proposition. One asks, then, did implementation of the proposition accelerate this decline?

Seven studies in our review described a pre-post policy assessment (16, 25, 28, 31, 49, 66, 74). These studies acknowledged the limitation of not statistically correcting for a time trend, i.e., not adjusting for changes in health outcomes over time that are occurring regardless of the policy, such as smoking rates declining due to smoking’s diminishing popularity. A methods chapter (68) referenced by one of these studies indicated that there is no conventional standard for selecting the starting time point for evaluating a time trend to subtract out its effect from a pre-post health evaluation. To illustrate this dilemma, see Figure 1 (1). As shown in the figure, California’s cigarette sales were already dropping precipitously prior to the implementation of Proposition 99 in 1988, which brought forth various anti-tobacco measures. To correct for the secular trend in cigarette sales, one could draw a regression line through pre–Proposition 99 cigarette sales trends and examine how much lower sales were post–Proposition 99. But in which years should the trend line start: in 1977, when the decline first becomes apparent, or from the first year of available data? Different choices of where to start the time trend line, and whether to reflect the trend as linear or nonlinear, could offer different estimates of the remaining policy effect.

**Figure 2:**
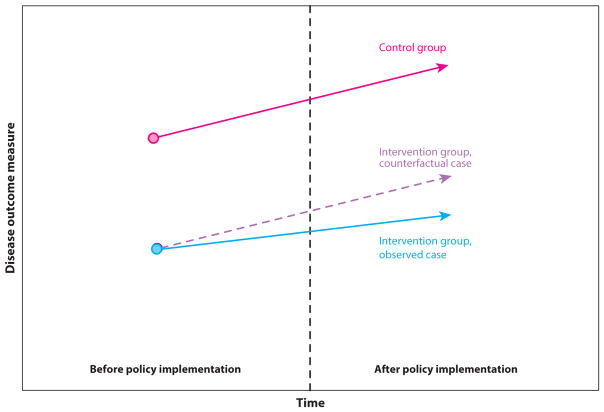
Illustration of difference-in-differences analysis. Two groups are followed over time, where the difference between the groups before the intervention affects one group is assumed to be the likely difference that would have been observed afterward between the groups if the intervention had not occurred (hence, the intervention group would have the *purple dashed line* trajectory without the intervention). The difference between the dashed intervention group point after the policy and the observed intervention group result is estimated to be the policy effect.

Six studies used an alternative to simple pre-post analysis to address the dilemma of correcting for time trends: the difference-in-differences (DD) approach (19, 35, 44, 52, 61, 71). Instead of comparing only health outcomes in a community before and after a policy implementation, a DD analysis (illustrated in Figure 2) additionally compares the change in health outcomes in the policy-exposed population to the simultaneous change in the health outcome in a comparable population unexposed to the policy. For example, to evaluate California’s tobacco control proposition, a DD analysis might compare smoking rates among Californians to smoking rates among Coloradans. Because Colorado did not implement similar tobacco control legislation during this time, the time trend in Colorado is assumed to project what would have occurred over time in the policy-affected California, had the policy not passed (Figure 2). In the DD analysis, the pre-post time point differences in smoking rates in California (smoking rate A1 prior to the policy and A2 after the policy) would be compared with the pre-post time point differences in smoking rates in Colorado (smoking rate B1 and B2, respectively). The difference in pre-post time point differences in smoking rates between the two states [the quantity (A2 − A1) − (B2 − B1)] would be attributed to the effect of the policy (see Figure 2). Hence, in accounting for a time trend, the DD approach accounts for unobserved confounders that might simultaneously affect both state populations similarly (e.g., national economic changes that could affect people’s ability to purchase tobacco).

Authors of the studies we reviewed performed the DD analysis by estimating the DD coefficient through a standard regression model:

$$Y_{i}=\beta_{1}\times {\mathrm{affected}}_{i}+\beta_{2}\times \mathrm{time}+\beta_{3}\times ({\mathrm{affected}}_{i}\times \mathrm{time}),$$

where for individual *i,* Y is the health risk factor or outcome of interest (e.g., Y = 1 if the person is smoking and Y = 0 if the person is not smoking), “affected” is a dummy variable for whether the person lives in a policy-affected or -unaffected community (e.g., 0 = Colorado, 1 = California), and “time” is a dummy variable for the policy period (0 = pre-proposition, 1 = post-proposition). Here, the β_1_ coefficient captures possible unobserved confounding differences between the populations in the two states prior to the policy; β_2_ accounts for the time trend in smoking, even if no policy had gone into effect; and β_3_ is the key coefficient of interest—the causal effect of the proposition on smoking rates. β_3_ is the DD in smoking rates and is equivalent to the quantity [(A2 − A1) − (B2 − B1)], or how much more smoking rates dropped in California than they did in Colorado.

The DD approach necessitates two critical assumptions: parallel trends and common shocks (23). The parallel trends assumption is that trends in the outcome of interest before the policy are similar in both the policy-affected and -unaffected communities. It is tested by running a second regression using the above equation, where time is a continuous variable (e.g., years) and the regression is isolated to the pre-policy period. If β_3_ is significant, the parallel trends assumption is violated, suggesting that the two states significantly differ in their pre-policy health outcome trends over time and presenting the need for a control group other than Colorado. If the β_3_ is insignificant, then Colorado is considered a valid comparator.

The common shocks assumption is, by contrast, untestable; it states that events occurring simultaneously or after the policy will affect both groups equally (e.g., the national economy may affect unemployment and associated cigarette sales but will affect Californians and Coloradoans equally). If, on the other hand, such shocks influence these states differently, this assumption is violated, and a source of confounding is potentially introduced. By taking the difference between the states’ data before and after the policy, the unobserved confounders that influenced Californians but not Coloradoans to pass the proposition are assumed to be “differenced out.” But, the DD approach cannot account for time-varying unobserved confounders (e.g., economic changes that affected one state more than the other), making the common shocks assumption often challenging to justify (68).

Although the common shocks assumption is one challenge to implementing the DD analysis, another challenge occurs when some subgroups of the policy-affected population are affected more than other subgroups. To study how a single subgroup might be most affected within a policy-affected group (such as a state), a triple differences or difference-in-difference-in-differences (DDD) specification is often used. For example, the DDD study in our review evaluated whether the mortality rates were lower in a state that expanded Medicaid insurance for the poor, as compared with mortality in a comparator state that did not expand Medicaid insurance (75). The authors defined subgroups of people *<*65 years old (i.e., eligible for Medicaid) versus the subgroup ≥65 years (i.e., ineligible and therefore unexpected to benefit from Medicaid expansion). Dummy variables for the subgroups (subgroup = 0 for *<*65 years old and 1 otherwise) and interaction terms were added to the standard DD regression equation, so the revised DDD equation read as follows:

$$Y_{i}=\beta_{1}\times {\mathrm{affected}}_{i}+\beta_{2}\times {\mathrm{subgroup}}_{i}+\beta_{3}\times \left({\mathrm{affected}}_{i}\times {\mathrm{subgroup}}_{i}\right)\;+\beta_{4}\times \mathrm{time}+\beta_{5}\times \left(\mathrm{time}\times {\mathrm{affected}}_{i}\right)+\beta_{6}\times \left(\mathrm{time}\times {\mathrm{subgroup}}_{i}\right)\;+\beta_{7}\times \left(\mathrm{time}\times {\mathrm{affected}}_{i}\times {\mathrm{subgroup}}_{i}\right),$$

where the β_7_ coefficient became the policy effect coefficient. The interaction terms in the equation filtered out changes in mortality among ≥65 years subgroups (assumed to be unrelated to the Medicaid expansion policy) and changes in mortality among all subgroups in the Medicaid-expansion state (assumed to be unrelated to the policy but related to other factors trending differently in the expansion versus control state).

One paper (36) highlighted that the DDD approach might be better than comparing pre-post policy mortality changes in the younger population to pre-post mortality changes in the older population within the same state because the latter approach accounts only for the state policy being studied, ignoring the effects of national policies on the outcome.

Two limitations or caveats accompanying the use of DD or DDD approaches were found in our review. First, one study found that DD or DDD formulations underestimated the standard error around the policy effect coefficient; the study’s researchers derived formulas to correct for this underestimation of the standard error (15). The second limitation was the risk of spillover effects (23), i.e., the possibility that policy-affected communities could influence policy-unaffected communities. Two studies we reviewed assessed for spillover by examining whether the population to whom the policy did not apply actually experienced a sudden change in outcomes at the time that the policy was implemented (63, 67).

## CONSTRUCTING A COMPARISON POPULATION

The DD approach requires the availability of a policy-unaffected community (a control group) that reasonably approximates the policy-affected community prior to policy implementation. Because identifying such a community is often difficult, the studies we reviewed adopted two approaches to create a control comparator population: a propensity score matching (PSM) approach or a synthetic control approach.

### Propensity Score Matching

Four studies used a PSM approach to form a comparator population for the policy-affected population (38, 45, 55, 71). All four had access to databases of individuals, some of whom were exposed to a policy of interest and others who were not. Yet, those unexposed often differed from exposed subjects in obvious ways, such as in income status or location. Therefore, the researchers developed a framework to select a subset of policy-exposed and -unexposed individuals who appeared similar on key covariates of concern. For example, one study evaluated whether a policy to provide social support to all elderly persons in a city reduced the rate of hospitalizations among the elderly; the researchers had two data sets on elderly adults living in a policy-affected city, and another sample of elderly adults living in unaffected cities (55). Because some subjects had vastly different incomes and health conditions (e.g., dementia) that may have been relevant to hospitalization rates, the authors used propensity scores to create subgroups of policy-affected and -unaffected persons who were more comparable with each other (55). A propensity score measures the estimated probability that individuals in a data set will experience policy exposure, given their observed features such as age, sex, income, and location. A propensity score is provided for each individual using logistic regression, in which the policy exposure (0 = policy unexposed, 1 = policy exposed in the data set) is regressed against observed covariates of interest. For example, in the study of social support among the elderly, the authors used characteristics such as income and dementia status to identify similar pools of elderly US adults, some who were exposed to the elderly support policy and others who were not. Each individual in the policy-exposed group was matched to an individual in the policy-unexposed group with the closest propensity score. During the matching process, a maximum allowable difference in scores (caliper distance) was set, and many individuals could be matched to one person or many-to-many depending on circumstances, as detailed in a cited review article on choice of matching algorithm (6).

Once the groups were constructed, the researchers checked whether the distribution of observable covariates (e.g., dementia scores) were balanced between the two groups (e.g., by plotting the distribution of covariates among the groups) and then performed a DD analysis. If pre-policy data were unavailable, the postpolicy outcomes between the matched populations could be compared in order to estimate the policy effect (4). Studies also stratified individuals into groups on the basis of propensity scores, used propensity scores as weights in regressions (weighting by so-called inverse probability of treatment), or simply adjusted covariates in regressions using propensity scores (23, 32, 33, 34). However, these latter approaches were reportedly less likely to correct for systematic differences in characteristics among populations than was PSM, according to methodological papers cited in the literature we reviewed (5, 7, 8).

All the studies identified the assumption of “strongly ignorable treatment assignment” as a key limitation to the PSM approach. According to the assumption, only observed covariates included in the regression equation used to estimate the propensity score can affect the probability of being exposed to the policy. Because unobserved confounders are not addressed by PSM, the approach may lead to biased policy effect estimates. For example, an unobserved variable such as local city government debt could affect whether a person’s city council passes the policy to provide elderly support. It may also influence the risk of hospitalization because debt could result in diminishing services such as transportation to primary care medical visits, which could increase the risk of hospitalization among elders that rely on the service to receive preventive health care. To check if bias may be present, one methodologic paper recommended estimating propensity scores and policy effects using multiple alternative control populations to determine if the policy effect remains consistent across alternative comparator populations (69).

Another key limitation of PSM is that inferences from the approach can be made only when both policy-unaffected and -affected individuals have nonzero probabilities of being in either group (known as the common support requirement). The approach cannot permit inferences on populations where investigators can find no matched person to reference in comparison.

### Synthetic Control Approach

Synthetic control analysis, another novel approach that rectifies the problem of not having an ideal control group, was used by one study to evaluate smoking rates in California after Proposition 99 (1). This method leverages insights from PSM but can be applied to aggregate populations (e.g., states) rather than to individuals to minimize the distance of observed covariates between the policy-affected and -unaffected population by constructing a new synthetic control population composed of weighted available control populations (e.g., a synthetic control California composed of a weighted Colorado, Nevada, and Oregon).

**Figure 3:**
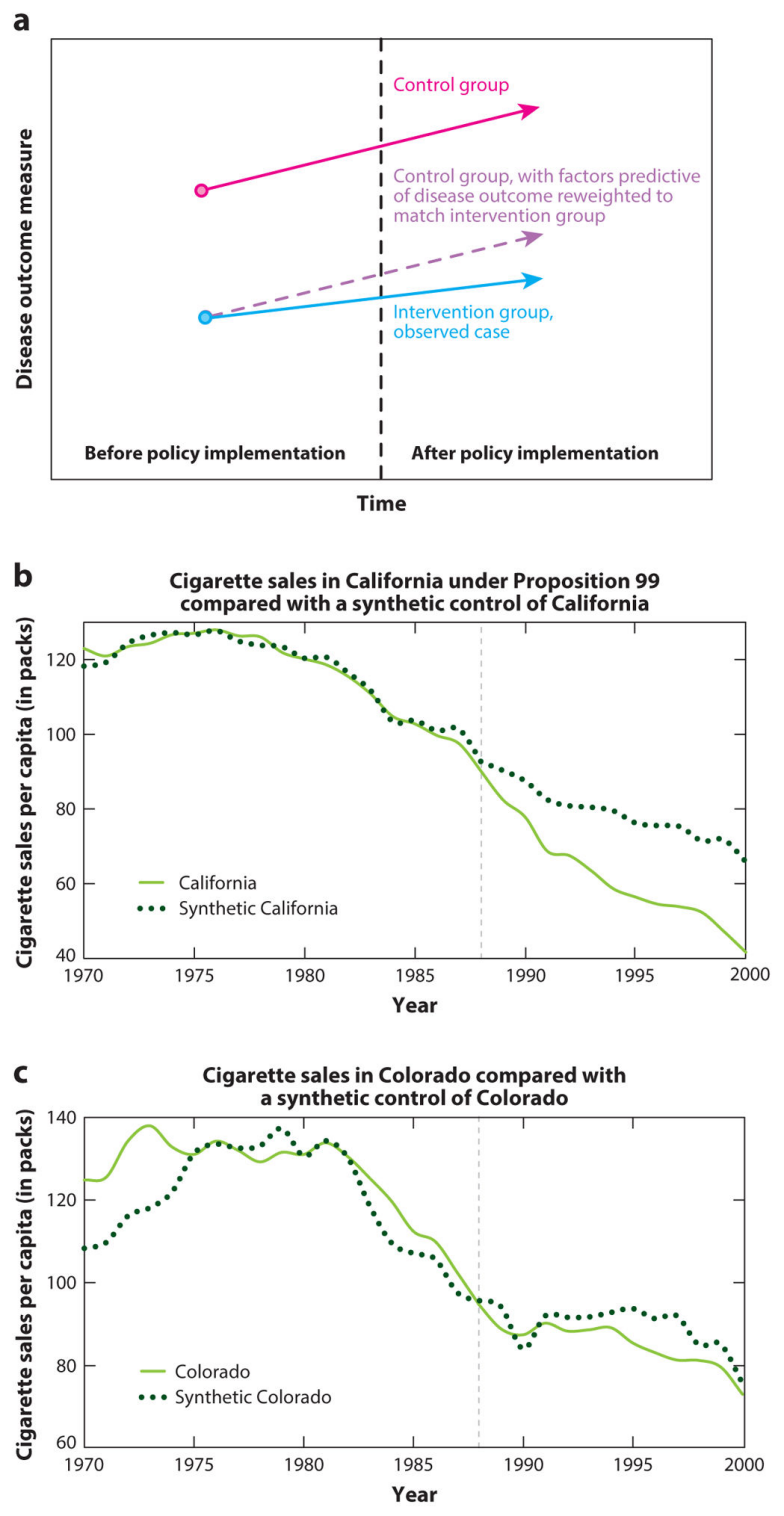
Synthetic control analysis. (*a*) Intuition behind synthetic control analysis. The control group populations are reweighted to match predictors of the health outcome in the intervention group, creating a synthetic intervention group. (*b*) Example of synthetic control analysis applied to tobacco smoking control policy in California. Independent reproduction of results from Reference 1. California, under the tobacco control regulation Proposition 99 (enacted in 1988, *gray dashed line*), is seen to deviate significantly from the synthetic California constructed as a weighted average of trajectories of other states, where the weights are determined by matching the predictors of tobacco smoking among the control states with the values of predictors of tobacco smoking in California. (*c*) Example of a placebo analysis. In this placebo test, states from the control pool are swapped out for California in the reweighting procedure, such that the weighting is reconducted as if one of the control states passed the new policy and the intervention state is now a control state. By doing all possible combinations of this swapping procedure, we can observe whether the observed trajectory of health outcomes in the intervention state is consistent or significantly inconsistent with all possible trajectories of health outcomes from all other states. Here, we see a comparison of Colorado’s smoking trajectory to a synthetic Colorado, which does not significantly differ from the observed trajectory.

The weights are constructed for treatment group *J* = 1 (e.g., California), and *J* = 2…*N* possible control groups (i.e., all states that did not implement a tobacco control policy), where *y_it_* is the outcome of the group *i* at time *t.* Weights *w_J_* are selected to $\text{minimize}\;(y_{11},x_{1})-\sum_{J=2}^{N}w_{J}(y_{J1},x_{J})$, or the difference in observed outcomes between the treated and weighted control groups in the pre-policy period, where *x_J_* are observable characteristics correlated to the outcome (e.g., a state’s poverty rate is correlated to its smoking rate). The policy effect is the difference between the observed outcome in treatment group *J* = 1 after introducing the policy outcome and the counterfactual outcome constructed from the control groups, estimated as $y_{12}-\sum_{J=2}^{N}w_{J}y_{J2}$. Figure 3 provides a conceptual illustration.

The factor weighting method in the synthetic control approach matches time-varying, policy-affected populations’ observed health outcomes with a set of their time-varying observed covariates not affected by the intervention (e.g., distributions of characteristics in each state such as poverty rates). A good pre-policy match should occur only if time-varying unobserved confounders are equally distributed among the exposed and synthetic control groups, making the method potentially less susceptible to bias than a standard DD approach.

In the study using this novel method (1), researchers conducted a placebo analysis (or falsification test) to measure changes in the health outcome when treating each control population as an intervention population (Figure 3). The placebo analysis involves redoing the analysis after substituting group *J* = 1 for each of the other policy-unaffected groups and having the treatment group enter among the control populations. The placebo analysis reveals what differences between the policy-affected and synthetic unaffected population are to be expected when no policy has been passed, owing to random variation; hence, one can evaluate whether the observed effect size in the policy-affected population is unusually large relative to the degree of random variation in the data set and therefore indicative of a real policy effect (Figure 3).

### Using Quasi-Random Variation

An RCT controls for unobserved confounding factors by randomizing individuals to either the treatment group or the placebo group; with sufficiently large sample size, unobserved factors should become equally dispersed among the two groups. The studies we reviewed were unable to simulate a perfect RCT, but we used various approaches to mimic randomization for population subgroups.

### Regression Discontinuity

**Figure 4:**
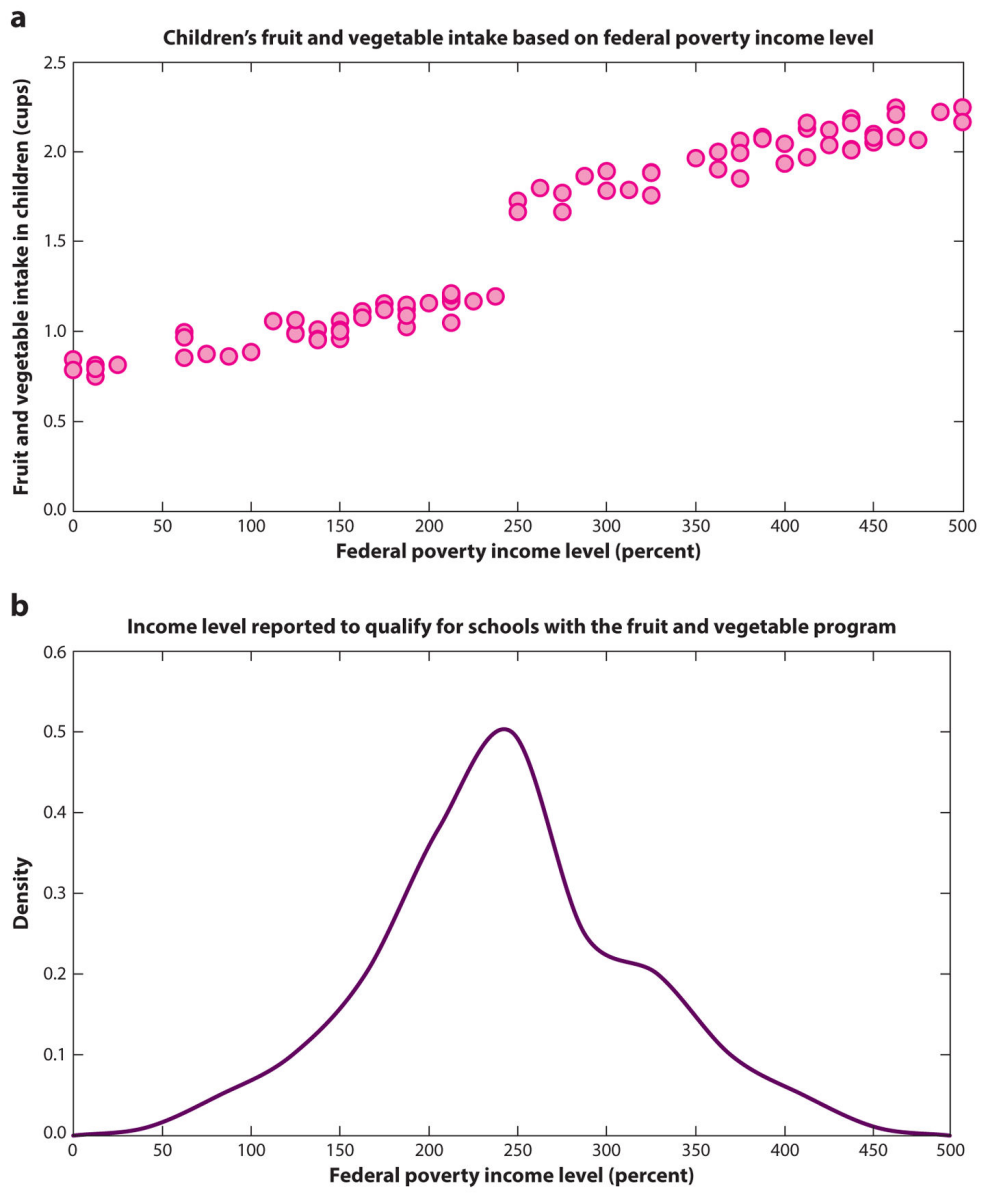
Regression discontinuity designs. Suppose we are evaluating the impact of a nationally subsidized, school fruit and vegetable program in which children at schools just below the neighborhood-level income threshold are included in program, whereas children that exceed the threshold are ineligible. In a theoretical data set, we can see in panel *a* that a discontinuity in fruit and vegetable intake appears on either side of the 250% federal poverty level cut point; however, in panel *b* we see that counties may be misreporting income levels so that children may qualify for a program-participating school because there is a notch showing abnormally high densities just above the 250% federal poverty line, suggesting misreporting of income levels to qualify for the program.

Four studies used regression discontinuity (RD) to perform policy analyses (30, 40, 60, 62). The RD approach is preferred when a control group is difficult to construct, for example when a national policy went into effect, leaving limited ability to compare outcomes across states (47). The approach compares people who minimally qualify for being affected by a policy to those who minimally missed qualifying and assumes that the randomness of being just slightly below or slightly above the threshold for being affected by the policy effectively randomizes people near the threshold to being affected or not (see Figure 4).

For example, one study found in our review used an RD approach to evaluate whether a national program that subsidized healthy foods among low-income schools improved students’ intake of fruits and vegetables (62). In this evaluation, populations of children at schools just below the neighborhood-level income threshold for being included in the program were compared with populations of children at schools where the neighborhood-level income minimally exceeded the threshold for eligibility.

A linear regression framework for estimating the policy effect was written as

$$Y_{i}=\beta_{0}+\beta_{1}\times {\mathrm{group}}_{i}+\beta_{2}\times \left({\mathrm{qualifier}}_{i}-\mathrm{threshold}\right)+\beta_{3}\times {\mathrm{group}}_{i}\times \left({\mathrm{qualifier}}_{i}-\mathrm{threshold}\right),$$

where for individual *i* (program-eligible children), Y is the outcome of interest (e.g., fruit and vegetable intake). “Group” is a binary variable referring to whether individual *i* is in the policy-affected or -unaffected group (1 if school receives the program, 0 otherwise), “qualifier” refers to the criteria that is used for policy eligibility (neighborhood income for individual *i* ) and “threshold” refers to the cut-off point for the policy (neighborhood income level to qualify for the program).

The above regression equation plots a line through the X–Y plot between the qualifier (income) and the outcome (fruit/vegetable intake), allowing for a shift in the slope and intercept of the line among people who are policy-affected versus those who are policy-unaffected (see Figure 4). If the policy effect is large, the regression will show a discontinuity at the point of threshold for policy eligibility. Although none of the studies we reviewed employed this design, the above study noted that the regression equation can also be estimated if the threshold for policy enrollment is not a sharp threshold cut point but rather a gradual cut point (e.g., a sliding scale for income eligibility), by using a fuzzy RD approach, which revises the regression equation to estimate probabilities of inclusion into the policy (3).

The studies we reviewed highlighted several important caveats to the RD approach. First, the RD approach does not perfectly mimic the generalizability of a randomized trial because it uses only data from individuals near the cut point for treatment (it estimates a local average treatment effect). It also focuses on a subset of the overall population and, because of the reduced sample size, has limited power compared with an RCT. Second, the threshold must be a truly randomizing factor (a point that is not itself related to the outcome) to account for the unobserved factors that differ between people who are affected versus those unaffected by the policy. For example, the fruit/vegetable program evaluation should apply to children whose schools are assigned on the basis of their neighborhood, not to those whose parents can select a school. Conceivably, parents conscious of eating more fruits and vegetables may select a program-participating school for their child if they have the ability to do so. To check for such manipulation in the RD analysis, researchers performed a density test, which plots the density of observations of the qualifier variable (i.e., neighborhood income) to ensure that the distribution does not have a notch (see Figure 4) near the threshold point to qualify for the policy. The presence of a notch would suggest, for example, that people are misreporting their income to qualify (53).

A falsification test was also commonly completed among the RD studies we reviewed. Doing so involves performing the RD analysis with an outcome variable that is determined before the policy effect takes place. If the discontinuity in the regression is present before the policy was proposed, then it is unlikely due to the policy.

Finally, studies also indicated that an RD approach could reasonably isolate policy effects from other treatment effects and prevent false attributions of causality only if the threshold for qualification was not being used by other programs. For example, the fruit/vegetable program threshold should not also be the threshold to qualify for other supplemental nutrition assistance.

### Instrumental Variables

**Figure 5:**
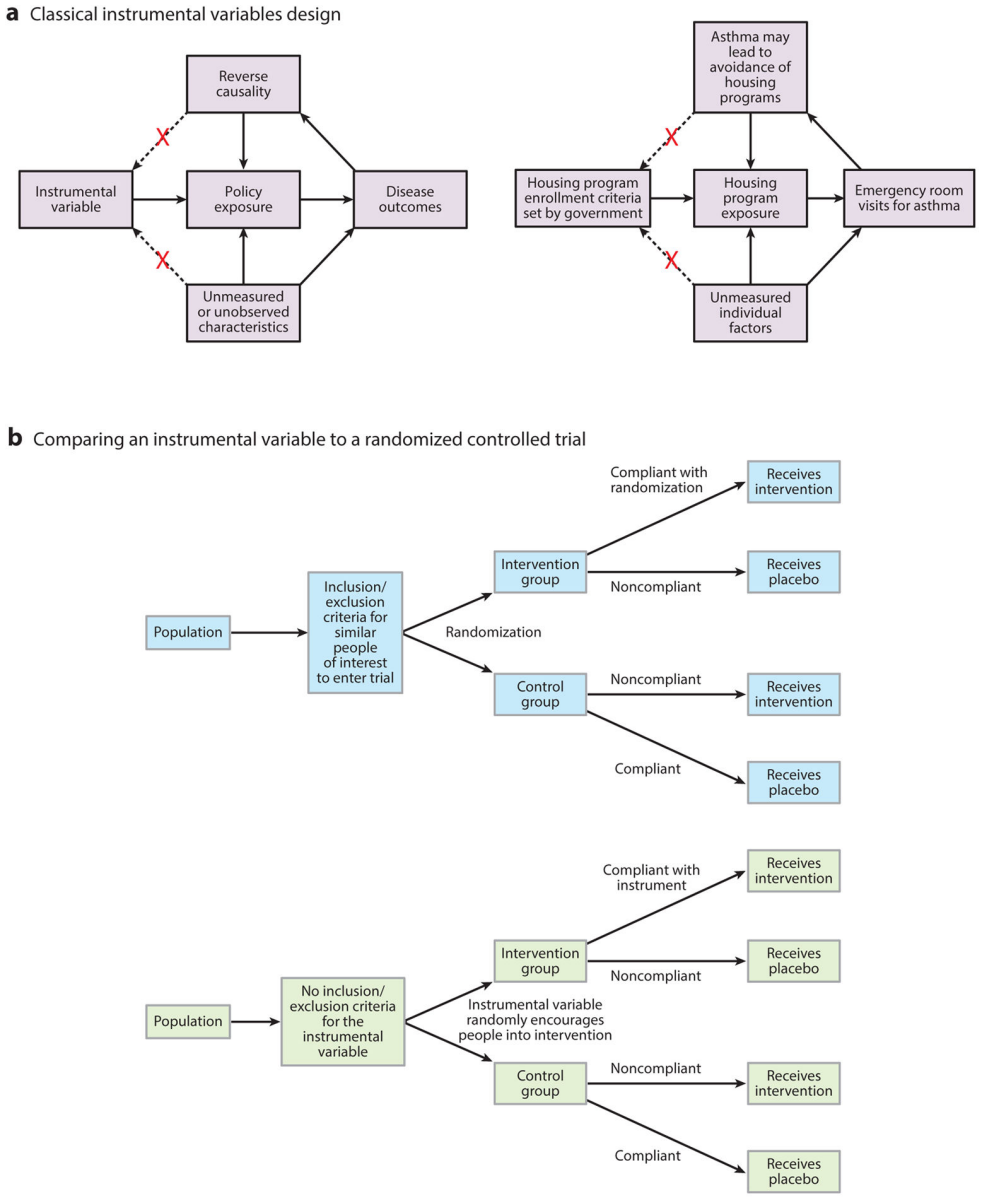
Principles behind instrumental variables analysis. (*a*) Classical instrumental variables design. (*b*) Comparing an instrumental variable to a randomized controlled trial reveals the problem of weak instruments. Weak instruments are those that randomly encourage members of the population to enter into a program or not, but many people can be noncompliant and not abide by the random encouragement.

Not all policies being studied have a clean threshold for affecting a subset of the population, which prevents investigators from using an RD approach. Many policies are diffused among individuals, households, and neighborhoods, with complex reasons for whether a population is affected by the policy or not. In such cases, 22 studies in our review used instrumental variable (IV) designs to estimate policy effects (11, 17, 18, 20, 21, 22, 26, 32, 33, 37, 39, 41, 42, 46, 50, 54, 56, 57, 58, 64, 73, 78). An IV is a factor that is related to the outcome of interest only by way of randomly encouraging or discouraging exposure to the policy (Table 1 lists key requirements for a valid IV, and Figure 5 presents a conceptual illustration).

One study investigated whether a federal program that subsidized food purchases for low-income Americans was inadvertently contributing to obesity by encouraging the purchase of sugar-sweetened beverages such as soda (12). Previous research had correlated participation in the program with higher rates of drinking sugar-sweetened beverages and higher obesity rates (14, 48). Yet, many unobserved confounders, such as living in a low-income neighborhood, may affect the likelihood of both participating in the nutrition program and consuming sugary drinks.

Because conducting an RCT and randomizing some, but not all, low-income Americans to the food subsidy program would be unethical, researchers used an IV approach. Study investigators selected a random factor that was uncorrelated with the outcome (sugary drink consumption) but encouraged or discouraged people to participate in the nutrition program. The random factor (IV) was whether a person lived in a state that required fingerprinting to sign up for the nutrition program. States that historically required fingerprinting, such as California and New York, discouraged some eligible persons from participating, whereas states that did not require fingerprinting, such as Nevada and Pennsylvania, were less discouraging to eligible persons.

The IV design mimics a matched-pair randomized trial, in which researchers randomized to the intervention or placebo group people who were similar. They then compared the sugary drink intake between the “encouraged” and “discouraged” groups, providing a local average treatment effect estimate of the nutrition policy on sugar drink consumption.

The IV analysis used a two-stage regression method, where the first-stage regression is

$$X=\beta_{1}\times \mathrm{instrument},$$

and the second stage is

$$Y=\beta_{2}\times (\mathrm{predicted}\;X\;\mathrm{values}\;\mathrm{from}\;\mathrm{first}\;\mathrm{equation})+\beta_{3}\times (\mathrm{control}\;\mathrm{variables}),$$

where X is the probability of policy exposure (e.g., being enrolled in the nutrition program), Y is the health outcome of interest (sugary drink consumption), and β_2_ identifies the estimated policy effect on health.

IV analysis has several requirements (Table 1). Most importantly, the selection of the instrument must truly provide random exogenous variation into the system, such that a third factor should not affect both the instrument and the outcome of interest and, in turn, the outcome should not be able to affect the instrument (reverse causality). Therefore, IV selection must be based on logical reasoning and knowledge of the policy and population. However, if the instrument is correlated with the error term, then the IV is likely invalid (13).

The selection of a weak instrument is another limitation. Although an instrument may encourage or discourage individuals in the program being studied, it may lack a definitive or strong influence. As shown in Figure 5, individuals can be noncompliant with the IV in responding to the random encouragement. A weak instrument degrades the reliability of policy effect estimates, potentially biasing estimates between the error terms in the two stages of IV analysis and leading to erroneous effect size estimates. However, the strength of an instrument can be tested; the F-statistic in the first-stage regression equation above should be larger than at least 10 (although some research suggests a conservative cutoff of 13) (76).

### Near-Far Matching

Two articles that faced the problem of weak IV used the newly devised strategy of near-far matching, which combines the benefits of PSM with IV techniques for analysis (51, 59).

**Figure 6:**
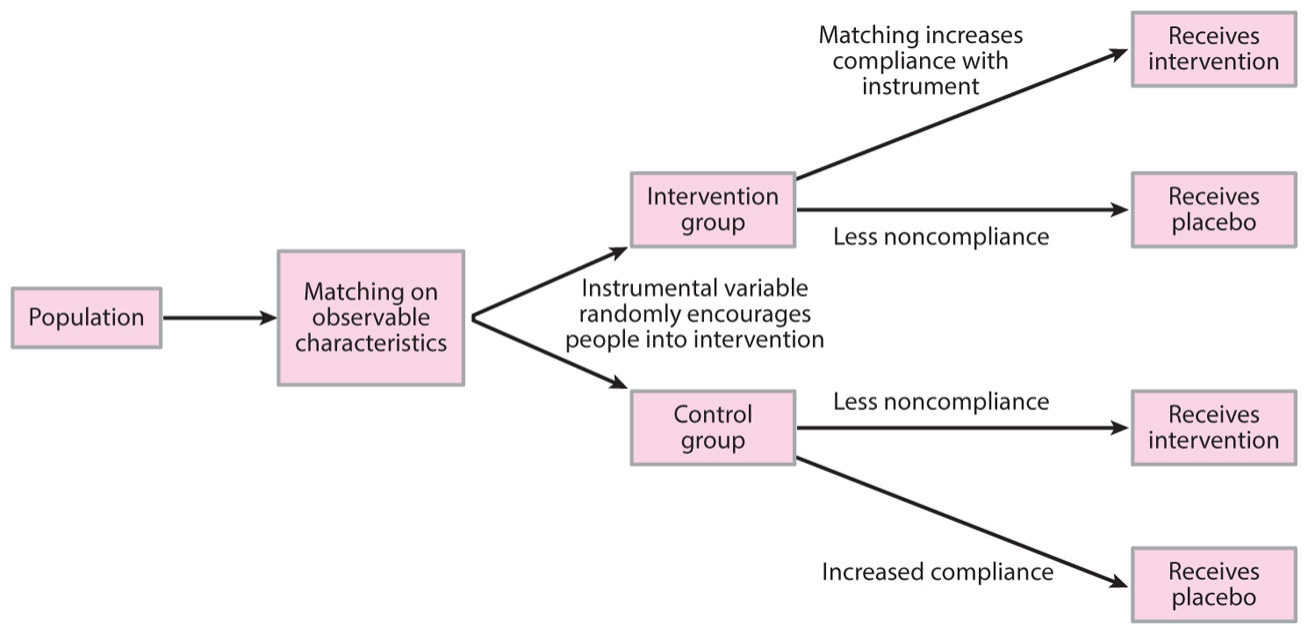
The intuition behind near-far matching. Compare with Figure 5***b***. As in a matched-pair randomized controlled trial, individuals are first matched on observable characteristics, which increases the probability that the influence of the instrumental variable will be to encourage persons into the policy of interest or not. As such, otherwise similar people (near to each other on observable characteristics) who receive different levels of encouragement into the program (far values on the instrumental variable) are likely to have different probabilities of enrolling into the program under study. A weak instrument is made stronger through this method.

Near-far matching also mimics a matched-pair RCT, as with standard IV analysis, but strengthens a weak instrument (see Figure 6) by simultaneously matching individuals in the data set to be as similar as possible (“near”) in their observed characteristics and as different as possible (“far”) in their values of an IV. For example, one study that examined birth outcomes attributable to a policy to fund new centers for treatment of high-risk pregnancy used distance to the hospital as an IV (51). However, the IV was weak (first-stage F-statistic test *<*10) because distance is only one of several considerations a mother makes when choosing where to give birth (51). Hence, the researchers used near-far matching to compare birth outcomes among otherwise-similar mothers for whom the IV was greatly different (i.e., they lived at very different distances to the hospital).

One advantage of near-far matching is its validity when the outcome of interest is dichotomous (e.g., the presence or absence of disease), unlike in classical IV approaches (9). In addition, by including an IV to effectively randomize people into the policy-exposed versus -unexposed groups, the near-far approach has the advantage of estimating causal effects even when a program is affected by confounding by indication, i.e., program enrollment is driven by an individual’s health condition (70). However, a key disadvantage of the near-far approach is that the matching may reduce the effective sample size of the assessment. Hence, there is a trade-off between how closely matches are performed (or how “near” to make matched individuals on observed covariates) versus how “far” to have them on the IV. The implications of alternative strategies for balancing these competing priorities are still under investigation (10).

## REINVIGORATING POLICY EVALUATION IN PUBLIC HEALTH

Our review reveals that public health researchers are increasingly gaining knowledge and experience in policy evaluation by borrowing study design approaches from the fields of econometrics, sociology, and political science—particularly for the common situation in which RCTs are not practically or ethically feasible. Our review indicates that, when a population is available to serve as a comparison group for a policy-affected population, the DD approach can offer advantages over a simple pre-post policy analysis focused on the affected population alone. Specifically, the DD approach can help correct for policy-unrelated time trends in the health outcome variable. However, the method requires assumptions, including the similarity of the comparison group’s health outcomes over time to the policy-affected population of interest. When an ideal comparison group is unavailable, the methods of PSM and, particularly for aggregate-level population statistics, synthetic control analysis may offer the opportunity to construct an artificial comparison population for the policy-affected population. When the policy is introduced or enforced in one population and not in a neighboring population, public health researchers can also use RD designs or IV designs (including the near-far matching variant for weak instruments) to estimate the local policy effect among people who are near the border zone of being potentially affected or unaffected by a policy.

None of these methods fully achieves the strength of an RCT in controlling for all unobserved covariates; however, they attempt to approximate such a trial as closely as possible for the populations of interest by minimizing bias in policy effect estimates from both observed and, to some extent, unobserved confounding. Most importantly, they offer opportunities to study policies more rigorously than simply performing the before and after comparisons that we found most common in the public health literature on policy evaluation. As we seek to better evaluate the health impacts of our public policies, we must also remember that the ecological fallacy—the risk of falsely attributing health effects to a policy because both policy passage and aggregate statistics are correlated owing to some confounder—must be balanced against the individualistic fallacy—that analysis of individual-level data are too often used to make inferences about whole populations (77). Large populations are not simply the sum of individual health outcomes but complex groups with interdependencies, producing the daunting task of identifying how best to analyze individuals, households, neighborhoods, countries, and whole societies. No single analytic approach can therefore replace an experienced, careful understanding of the population being studied, the policy being examined, and how the two interrelate.

Increasing our understanding of policy evaluation approaches may enhance our ability to prospectively collect data needed to perform valid analyses of large public policy interventions that affect public health. For example, when new legislative measures are first being proposed, we can anticipate which types of data to collect from both intervention and control groups to prospectively evaluate enacted policies through the methods outlined here. We hope that our increasing consciousness and planning will advance us to an era where observational research on large-scale policies that theoretically affect public health is considered highly valid and feasible and not simply an exceptional natural experiment evaluated through questionable correlations of aggregate data.

## Figures and Tables

**Table 1:** Key requirements for a valid instrumental variable (see also Figure 4)

Requirement	Description
Exclusion restriction	Any effect of the instrument on health must be mediated by exposure to the policy of interest
Exogenous	The instrument must be randomly distributed and uncorrelated with the unobserved or unmeasured characteristics of individuals
Meaningful effect	The instrument must reliably predict the policy exposure (a strong instrument)
Monotonic	The effect of the instrument on the policy exposure is not smaller than the exposure that would occur without the instrument
Stable unit treatment value	The value of the policy variable and the relationship between the policy variable and health outcome in one individual must not be affected by variations in these factors among other individuals
